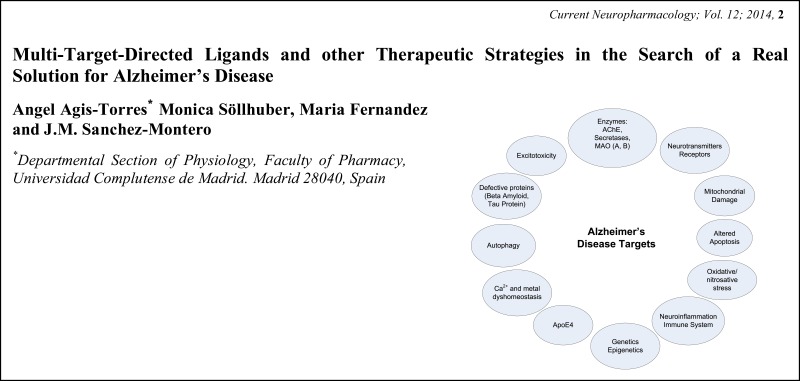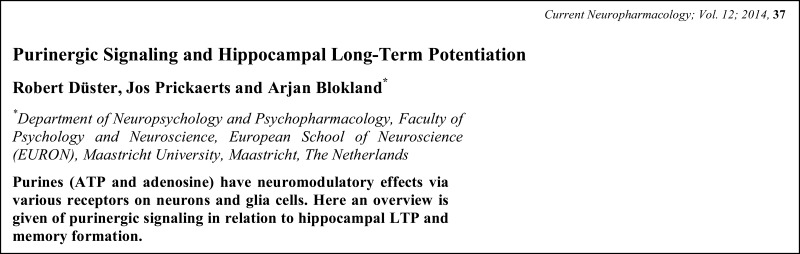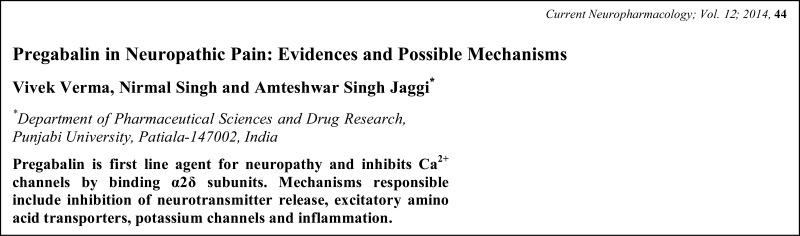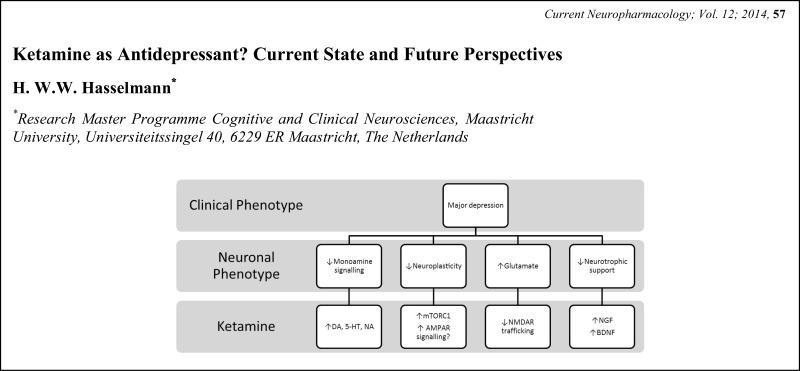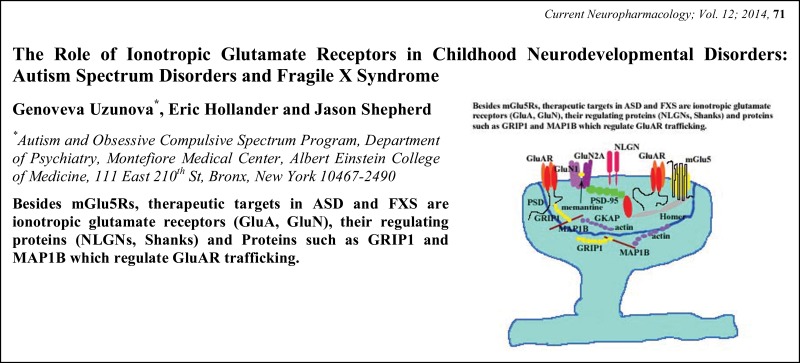# Graphical Abstracts

**DOI:** 10.2174/1570159X1201140117155544

**Published:** 2014-01